# Effect of COVID-19 pandemic on well child follow-up visits and vaccination: A Single unit experience from Turkey

**DOI:** 10.12669/pjms.41.1.9689

**Published:** 2025-01

**Authors:** Merve Tosyali, Feyza Koc

**Affiliations:** 1 Merve Tosyali, MD Division of Social Pediatrics, Department of Pediatrics, Ege University Children’s Hospital, Ege University Faculty of Medicine, Ege University, Bornova, Izmir, Turkey; 2 Feyza Koc, MD Associate Professor, Division of Social Pediatrics, Department of Pediatrics, Ege University Children’s Hospital, Ege University Faculty of Medicine, Ege University, Bornova, Izmir, Turkey

**Keywords:** COVID-19, Child Health, Vaccination

## Abstract

**Objective:**

The aim of the study was to examine the effects of the COVID-19 pandemic on frequency of well-child follow-up visits and immunization rate in Turkish tertiary reference hospital’s Well-Child Care Outpatient Clinic.

**Methods:**

Children aged one month to 18 years who presented to the Well Child Care Outpatient Clinic of a tertiary referral hospital in Turkey for child health follow-up and immunisation were included in the study. Children with chronic diseases or children who needed to be immunised with a different scheme due to their special conditions were not included. In our cross-sectional study, the cases were categorised into two distinct groups based on application dates; those who applied between September 1, 2018, and March 1, 2020, were assessed during the pre-pandemic period, while those who applied between March 11, 2020, and November 1, 2021, were assessed during the pandemic period. The pandemic and pre-pandemic numbers of children admitted to our clinic, their sociodemographic characteristics, and immunizations were compared.

**Results:**

The pre-pandemic number of children admitted for child health follow-up was 9368; the pandemic number was 3830. In the pandemic, 21.8% of children were vaccinated. Although the number of children admitted to our clinic decreased during the pandemic period, vaccination rates increased significantly (p = 0.0036).

**Conclusion:**

The COVID-19 pandemic has had a significant impact on healthcare. In addition to the effects that emerged during the pandemic period, it is predicted that there will be many reflections in the post-pandemic period, especially affecting the vulnerable population. Although our study was conducted in a single centre, it is of great importance in terms of revealing the efforts made by healthcare workers in the field of child health follow-up and vaccination during the pandemic period and emphasising that primary healthcare services should continue.

## INTRODUCTION

Coronavirus-19 (COVID-19) first appeared in the Wuhan province of China in late 2019, and then the World Health Organization (WHO) declared a pandemic on March 11, 2020.^1^ After this date, precautions began to be taken all over the world because the infection was very contagious and its consequences were unpredictable. Some of the precautions to reduce contact taken during the pandemic included implementing curfews, encouraging working from home, closing schools, and restricting travel. There have also been significant changes in healthcare services, such as outpatient clinics being closed and only emergency cases being admitted to the hospital. As a result of all this, access to routine health services, including immunization services, has been limited during the COVID-19 pandemic.^2^,^3^

Although children have a low mortality rate as a result of COVID-19 infection, they have been among the groups most affected by the pandemic.In addition to the above-mentioned contact precautions, families’ fear of transmission of infection from hospitals or public transport, as well as the economic difficulties caused by the pandemic, prevented children from accessing basic health services.^4^-^6^ WHO has reported that more than 80 million children under the age of one have been affected due to the disruption of routine childhood immunization services during the pandemic, especially in low-income countries.^7^ Disruptions in immunization services may increase vaccine-preventable infections and have serious adverse effects on childhood morbidity and mortality related to these diseases. 6 During the pandemic, WHO issued a guideline emphasizing that child vaccination programs should be maintained with special measures. 7 Despite this, studies from many countries, especially developing countries, have clearly shown that follow-up visits of healthy children have been interrupted and vaccination rates have declined.^8^-^13^

In our country, the Ministry of Health, similar to the WHO, has reported that vaccination, which is a routine health service in family medicine centers, should be continued.^14^ During the COVID-19 pandemic period, we continued child health follow-up and vaccination with a rapid adaptation to personal protective equipment and good hygiene practices, taking physical distance measures for infection control in our clinic. However, during the pandemic, although there has been a significant decrease in child health follow-up visits and vaccination rates in our country due to severe health system restrictions and physical distancing measures implemented to mitigate the pandemic, there is no comprehensive study examining this. The aim of the study was to examine the effects of the COVID-19 pandemic on frequency of well-child follow-up visits and immunization rate in a Turkish tertiary reference hospital’s Well-Child Care Outpatient Clinic.

## METHODS

In our cross-sectional study, the cases were categorised into two distinct groups based on hospitalization dates; those who visited between September 1, 2018, and March 1, 2020, were assessed during the pre-pandemic period, while those who applied between March 11, 2020, and November 1, 2021, were assessed during the pandemic period. Sociodemographic data such as age, gender, date of birth, time of birth, age, occupational status and educational level of parents, marital status, family income, and vaccine administration information in the relevant period were obtained retrospectively from electronic patient files. Babies born before 37th gestational week are defined as preterm and babies born after 37th gestational week are defined as term.

The study included children aged one month to 18 years who presented for child follow-up at the Well Child Care Outpatient Clinic of a tertiary referral hospital in Turkey. Only children between the ages of one month and 18 years who applied to our outpatient clinic for child health follow-up and vaccination were included in the study; children with chronic diseases or children who needed to be vaccinated with a different scheme due to their special conditions were not included. Also, whose parents did not consent to enrollment in the study were excluded.

Vaccinations administered to children were categorized as routine and non-routine vaccines. In our country, vaccines such as Bacillus Calmette-Guérin vaccine (BCG), Hepatitis B, DTaP+IPV+Hib (diphtheria, tetanus, acellular pertussis, inactivated poliovirus, Haemophilus influenza type b), Pneumococcal conjugate vaccine (PCV-13), Oral poliovirus (OPV), Measles-Mumps-Rubella (MMR), Varicella, and Hepatitis A are included in the national vaccination schedule and are defined as routine vaccines. Vaccines such as Rotavirus, Meningococcal Serogroups A, C, W, and Y (MenACWY), Meningococcal Serogroup B (Men B), Influenza (inactivated), and Human Papillomavirus (HPV) are not included in the national vaccination schedule and are defined as non-routine vaccines. In our country, routine vaccines included in the national vaccination scheme are mostly administered free of charge in family health centres. However, in our child health follow-up outpatient clinic, child health follow-up visits, routine and non-routine vaccination services, newborn endocrine and metabolic screening tests, examinations, as well as breastfeeding counselling and family counselling services are regularly carried out for all children aged 0-18. So, we inform the family about non-routine vaccines so that they can be administered as early as possible. If the family wants to have this vaccine, we provide it for a fee and administer it in our outpatient clinic. We provide intensive information sharing with parents about the growth of a baby from birth and what needs to be done for the healthy development of the child. We build a reliable bond between the child, the parents and the medical staff.

The number of children admitted to our clinic, their sociodemographic characteristics, and the vaccines administered were compared in the pandemic and pre-pandemic periods. The study was conducted according to the guidelines of the Declaration of Helsinki. The study was conducted according to the guidelines of the Declaration of Helsinki.

### Ethical Approval:

This study was approved on 12 May 2022 by the Ege University Medical Research Ethics Committee (Approval No. 22-5T/27, dated May 12, 2022). Informed consent was obtained from all subjects involved in the study.

### Statistical Analysis:

Statistical analyses were performed on SPSS 25.0 software (IBM Corp., Armonk, NY, USA). The descriptive statistics of the data obtained from the research used mean and standard deviation for numerical variables’ frequency and percentage analysis for categorical variables. Chi-square and Student t-tests were used for the relationship between variables. According to Fisher’s normal distribution test, the data were found to fit the normal distribution. The results are given as numbers and percentages. For all analyses, p < 0.05 was considered significant.

## RESULTS

In our study, it was determined that while the number of children admitted for child health follow-up was 9368 in the pre-pandemic period, which decreased to 3830 in the pandemic period. Demographic characteristics of the children in our study are shown in Table-I.The gender distribution of the participants was similar in both periods. In the study, statistically significant differences were found in children admitted during the pre-pandemic and pandemic periods in terms of time of birth, parental education, and occupational status (*p*<0.05). While the birth times of 78% of the children in the pre-pandemic period were determined to be mature, the birth times of 80% of the children during the pandemic period were determined to be mature (*p* = 0.011).

**Table-I:** Demographic data of the children included in the study.

	Pre-pandemic period n=9368	Pandemic period n=3830	p-value
***Age***			*p* = 0.194
1-24 months	7026 (75)	2831 (73.9)	
>24 months	2342 (25)	999 (26.1)	
***Time of birth***			*p* = 0.011
Mature	7307 (78)	3064 (80)	
Premature	2061 (22)	766 (20)	
***Gender***			*p* = 0.845
Male	4852 (51.8)	1999 (52.2)	
Female	4515 (48.2)	1831 (47.8)	
***Mother’s education***			
1-8 years	843 (9)	805 (21)	*p*< 0.001
9-12 years	1113 (11.9)	603 (15.7)	
University	7412 (79.1)	2422 (63.3)	
***Father’s education***			
1-8 years	1826 (19.5)	1132 (29.6)	*p*< 0.001
9-12 years	1256 (13.4)	905 (23.6)	
University	6286 (67.1)	1793 (46.8)	
***Occupational status of mother***			*p*< 0.001
Housewife	2904 (31)	996 (26)	
Working	6464 (69)	2834 (74)	
***Occupational status of father***			*p*< 0.001
Unemployed	1030 (11)	573 (15)	
Working	8338 (89)	3257 (85)	

(n, %).

The educational level of mothers and fathers who brought their children to our outpatient clinic for child health follow-up and vaccination in the pre-pandemic period was higher than the educational level of mothers and fathers who applied during the pandemic period (p<0.001). In the pre-pandemic period, 8338 of the fathers of the children (89%) were working, but this number was determined to be 3257 (%85) during the pandemic period (*p*<0.01).

In the pre-pandemic period, 16.8% of the children who came for follow-up of healthy children were vaccinated, while 21.8% were vaccinated during the pandemic period (p= 0.0036) (Table-II). In addition, in our study, it was found that 667 (42%) of the vaccines administered to children in the pre-pandemic period and 493 (58.9%) in the pandemic period were routine vaccines (p<0.001). When the non-routine vaccines administered were evaluated, it was observed that meningococcal vaccine was administered most frequently in both periods, and rotavirus vaccine ranked second.

**Table-II:** Evaluation of children to follow-up visits and vaccine applications.

	Pre-pandemic period	Pandemic period	p-value
The number of children admitted to well-child follow-up visits, n	9368	3830	p = 0.0036
The number of children applying for vaccination, n (%)	1589 (16.9)	837 (21.8)	
Administered vaccines, n (%)			
Routine vaccines	667 (42)	493 (58.9)	p<0.001
Non-routine vaccines	974 (61.3)	366 (43.7)	

The demographic characteristics of the vaccinated children are shown in Table-III. It was found that the vaccinated children during the pre-pandemic and pandemic periods had statistically significant differences had statistically significant differences in terms of age, time of birth, parental education, and occupational status (Table-III). During the pandemic, 93.6% of children vaccinated were under two years of age, and this increase was statistically significant when compared to the ages of children vaccinated in the pre-pandemic period (p = 0.029). When the gender distribution of the patients who applied for vaccination was analysed, it was found that there was no significant difference between the pre-pandemic period and the pandemic period. While 38.8% of children vaccinated during the pandemic period had a history of premature birth, this rate was 21% in the pre-pandemic period (p <0.001). In addition, the unemployment rate of parents of children vaccinated during the pandemic period was higher than in the pre-pandemic period (p <0.001).

**Table-III:** Demographic data of the vaccinated children.

	Pre-pandemic period (n=1589)	Pandemic period (n=837)	p-value
***Age***			*p* = 0.029
1-24 months	1171 (73.7)	806 (96.3)	
>24 months	418 (26.3)	31 (3.7)	
***Time of birth***			*p*< 0.001
Mature	1255 (79)	512 (61.2)	
Premature	334 (21)	325 (38.8)	
***Gender***			*p* = 0.127
Male	815 (51.3)	402 (48)	
Female	774 (48.7)	435 (52)	
***Mother’s education***			
1-8 years	254 (15.9)	100 (11.9)	*p*< 0.001
9-12 years	185 (11.6)	294 (35.2)	
University	1150 (72.5)	443 (52.9)	
***Father’s education***			
1-8 years	197 (12.4)	57 (6.8)	p<0.001
9-12 years	296 (18.6)	179 (21.4)	
University	1096 (69)	601 (71.8)	
***Occupational status of mother***			*p* = 0.001
Housewife	594 (37.4)	502 (60)	
Working	995 (62.6)	335 (40)	
***Occupational status of father***			*p* = 0.001
Unemployed	113 (7.1)	92 (11)	
Working	1476 (92.9)	745 (89)	

(n, %).

## DISCUSSION

The COVID-19 pandemic is known to have had a significant impact on healthcare services going forward.^6^ Child health follow-up visits are the setting where childhood immunisations are routinely administered. Our study shows that during the pandemic period, admissions to our clinic for child health follow-up and vaccination have decreased significantly compared to the pre-pandemic period. However, it is noteworthy that the cases, mostly children under two years of age with an intensive vaccination plan, presented to our outpatient clinic on the scheduled vaccination dates. Although the number of patients visiting our outpatient clinic decreased during the pandemic period, it was observed that vaccination rates increased significantly compared to the pre-pandemic period. This is the most important result of our study. This success is due to the fact that our outpatient clinic is the only paediatric outpatient clinic serving outside the paediatric emergency department in our hospital; that we continue basic child health follow-up services during the chaotic pandemic period by complying with isolation measures, paying attention to distance and hygiene rules and providing sufficient personnel; the fact that our children and parents who are coming to regular follow-up may have been informed about the importance of child health follow-up and immunisations before the pandemic.

In addition to its global impact on health services, the COVID-19 pandemic has changed daily life in many ways. WHO reported some disruption to essential health services in 90 per cent of 105 countries, with routine immunisation services among the most frequently disrupted.^2^,^15^ During this period, strict home quarantine to reduce viral exposure and the closure of health centres other than emergency departments significantly disadvantaged newborns and young children. Parents have stopped attending routine paediatric health surveillance care and immunisation appointments to protect their children.^16^-^18^ This trend has continued despite the limited reopening of doctors’ surgeries. This has resulted in postponement of childhood immunisations. In a study conducted in South Africa, it was found that there was a decrease of more than 50% in basic health follow-up visits of children under the age of five.^19^ Similarly, studies conducted in other countries of the world have reported a decrease in child health follow-up visits.^2^,^3^,^20^,^21^ Although our outpatient clinic was open to ensure that basic paediatric health services were not interrupted by taking isolation measures into account, our study showed that the number of children admitted to our outpatient clinic during the pandemic period decreased by 59% compared to the pre-pandemic period. Even though access to healthcare services is limited, it is of great importance that healthcare professionals make efforts to carry out routine follow-ups in healthcare centers.

The COVID-19 pandemic not only caused disruption of child health follow-up visits, but also caused a decrease in childhood vaccination rates, which is predicted to lead to a great danger to child health all over the world.^8^,^10^ A study conducted in Pakistan reported a 51% decrease in vaccination rates in children under two years of age.^11^ In another study, Ackerson et al. reported that the total dose of routine vaccines administered to aged 0 to 18 was reduced compared to the pre-pandemic period in California.^22^ In a study examining the vaccines given to children during the pandemic period in detail, it was reported that MMR vaccinations decreased between 25.6% and 73.6% compared to previous years.^21^ Similar to other studies, we found that the number of children vaccinated in our centre decreased by approximately 50% compared to the pre-pandemic period. However, it was determined that the vaccination rate of children who visited well-child follow-up during the pandemic period increased compared to the pre-pandemic period, which is a pleasing finding. The results of our study reveal single-center data, but the most important reason for the increase in the number of vaccinated children was the great efforts made by our healthcare personnel. During the pandemic period, the information given to the families by the Ministry of Health through the media about the importance of vaccination and that it should not be interrupted may also have had contributed to this.^14^

Another prominent result of our study was that the rate of vaccination with non-routine vaccines, such as meningococcal vaccines and rotavirus vaccines, was higher during the pandemic period compared to the pre-pandemic period. It is thought that the detailed information we provided to parents about vaccines in our outpatient clinic services before the pandemic may have been effective in this result.

As a result of the economic and social effects of the COVID-19 pandemic, the working order has changed in many business sectors, some have reduced the number of employees, and some have switched to working from home. Unemployment rates increased in our country during this period. As a reflection of this, when the demographic data of the cases who applied to our outpatient clinic for child health follow-up were examined in our study, it was observed that the level of parental education was lower during the pandemic period compared to the pre-pandemic period, and the unemployment level of fathers was much higher during the pandemic period. Similarly, in the current literature, it has been reported that the fear of infection of parents with high education level and their frequent transition to working from home can be seen as a reason why they do not bring their children to the hospital for routine follow-up and vaccination.^23^ In a comprehensive study examining the benefit-risk ratios of maintaining childhood vaccination programmes during the COVID-19 pandemic in African countries, it was shown that while there is heterogeneity in the implementation of and compliance with prevention and control measures for COVID-19, the benefits of maintaining childhood vaccination far outweigh the risks of excessive SARS-CoV-2 infections acquired during vaccination visits, especially for vaccinated children. 24 As determined in our study, each country should have a well-coordinated action plan to ensure that child health follow-up visits and vaccination rates do not decrease even in epidemic situations. Healthcare workers can contribute to this goal by calling families when routine follow-up and vaccination dates are due and calling infants and children to the health centre where distance and hygiene rules are followed and appropriate examination conditions are provided.

### Strength of the Study:

Although conducted in a single center, our study holds great importance as it highlights the efforts made by healthcare professionals during the pandemic and emphasizes the need for the continued provision of general healthcare services. This is also the first study conducted on this subject in our country. The results we have obtained can be a guide to determine the strengths and weaknesses of the strategies implemented in the health system during the pandemic process and to guide the measures to be taken for similar situations that may occur in the future.The COVID-19 pandemic has had a significant impact on healthcare. In addition to the destructive effects that emerged during the pandemic period, it is predicted that there will be many reflections in the post-pandemic period, especially affecting the vulnerable population.

Although our study was conducted in a single centre, it is of great importance in terms of revealing the efforts made by healthcare workers in the field of child health follow-up and vaccination during the pandemic period and emphasising that primary healthcare services should continue.

### Limitations:

This cross-sectional study does not reflect the entire population due to the sample size and timing. It cannot be generalized, as parents who do not have access to health care were not included. The data provided does not represent the standard vaccination rates across the country.

## CONCLUSION

In the case of the COVID -19 pandemic, it is predicted that epidemics will have many repercussions affecting especially the vulnerable population in the post-epidemic period, as well as the devastating effects on the health services of societies. Therefore, policy makers and experienced health personnel should develop strategies to ensure that basic health services, such as child health follow-up visits and vaccination practices, continue without interruption during possible future outbreaks. Determining effective and feasible monitoring and vaccination strategies that are planned before outbreaks occur is the only way out to prevent possible disasters.
